# Three‐Dimensional Mesh Recovery from Common 2‐Dimensional Pictures for Automated Assessment of Body Posture in Camptocormia

**DOI:** 10.1002/mdc3.13647

**Published:** 2023-01-24

**Authors:** Robin Wolke, Olga Gavriliuc, Oliver Granert, Günther Deuschl, Nils G. Margraf

**Affiliations:** ^1^ Department of Neurology Kiel University, Universitätskrankenhaus Schleswig‐Holstein, Campus Kiel Kiel Germany; ^2^ Department of Neurology State University of Medicine and Pharmacy "Nicolae Testemitanu" Chisinau Moldova

**Keywords:** camptocormia, posture, automated assessment, Parkinson, axial‐postural disorders

## Abstract

**Background:**

Three‐dimensional (3D) human body estimation from common photographs is an evolving method in the field of computer vision. It has not yet been evaluated on postural disorders. We generated 3D models from 2‐dimensional pictures of camptocormia patients to measure the bending angle of the trunk according to recommendations in the literature.

**Methods:**

We used the Part Attention Regressor algorithm to generate 3D models from photographs of camptocormia patients' posture and validated the resulting angles against the gold standard. A total of 2 virtual human models with camptocormia were generated to evaluate the performance depending on the camera angle.

**Results:**

The bending angle assessment using the 3D mesh correlated highly with the gold standard (*R* = 0.97, *P* < 0.05) and is robust to deviations of the camera angle.

**Conclusions:**

The generation of 3D models offers a new method for assessing postural disorders. It is automated and robust to nonperfect pictures, and the result offers a comprehensive analysis beyond the bending angle.

The term *camptocormia* refers to a severe, pathological, nonfixed forward flexion of the trunk greater than 30 degrees that occurs during the course of several neurological diseases and means a severe reduction in quality of life due to back pain, reduced mobility and autonomy.^1^ The usual assessment of this flexion relies on the manual assessment of photographs as defined in a consensus statement.^2^ Up to date the manual method of angle assessment produced the only empirical cutoff criterion for camptocormia. There have been attempts to automate and objectify the measure of the posture in camptocormia with computer‐vision methods. In the consensus of clinical experts, the use of the total‐camptocormia‐angle method assessed by the so‐called malleolus method is recommended to quantify the camptocormia bending angle.^2^ Deviating from this, the method by Shin et al measured the angle between the lateral malleolus, the estimated hip joint, and shoulder positions using the 2‐dimensional (2D) posture estimation framework “OpenPose,” reaching a promising validity of their approach.^3^ Another study that used a comparable measuring technique was based on the “Kinect” depth camera that enriches 2D–RedGreenBlue (RGB) images with depth estimation data and led to promising results as well.^4^ In contrast to the aforementioned studies, this study aims to evaluate the use of state‐of‐the‐art 3‐dimensional (3D) model generation (3D mesh recovery) for the documentation of the camptocormia angle and follows the current assessment guideline in an automated fashion. Moreover, a 3D model of the patient's posture offers a visually appealing clinical impression and holds potential for advances in the remote counseling of patients. This new approach, which has not yet been evaluated, will be used to compare it with the current gold standard in the accuracy of angle detection and to check whether there is greater robustness, especially with regard to unwanted rotation of the subject relative to the camera.

## Methods

**FIG. 1:**
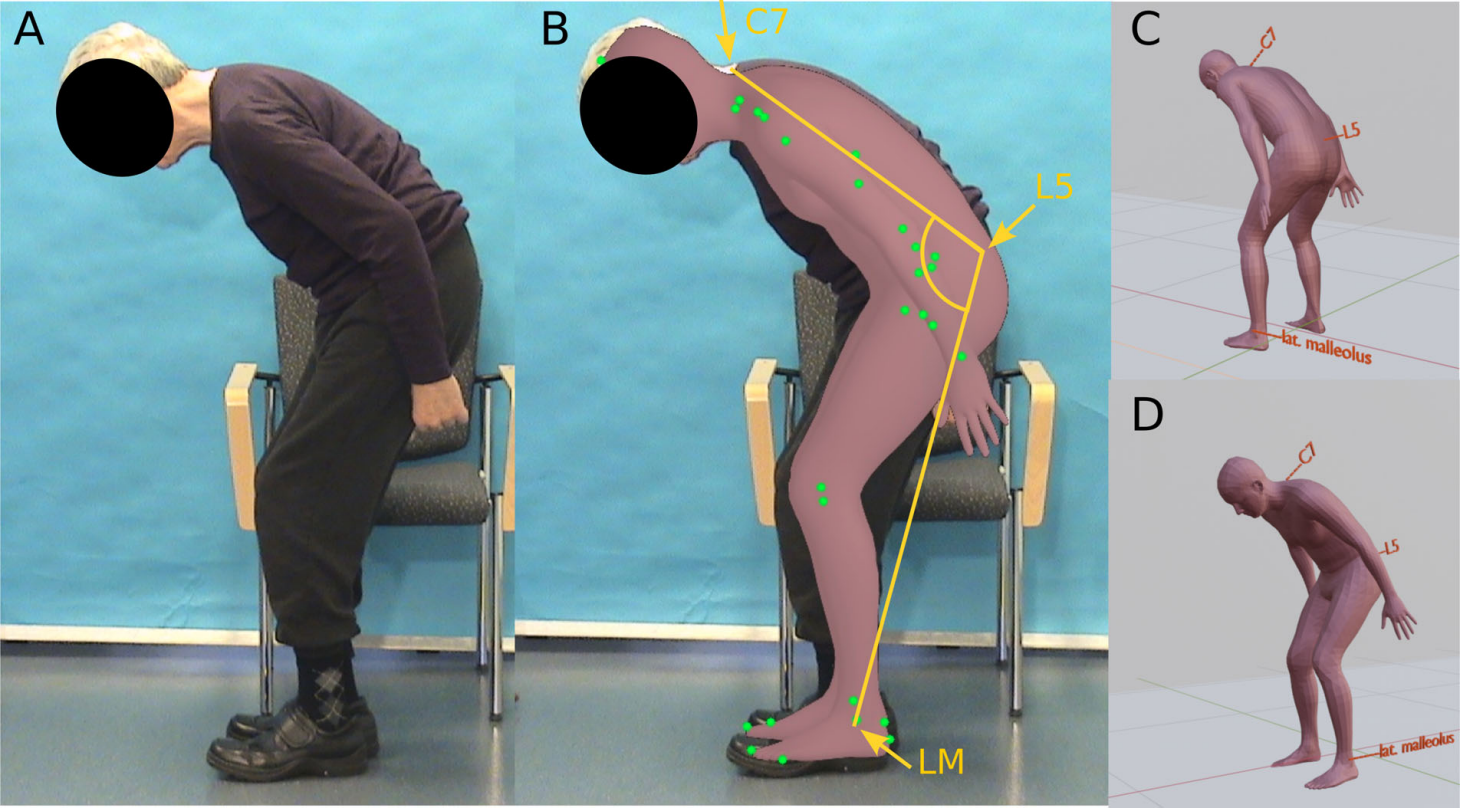
(**A**) Example of a patient with Parkinson's disease with camptocormia. (**B**) The 3‐dimensional mesh is overlayed, and the green dots mark the automatically identified key points for the pose estimation algorithm used prior to mesh fitting. Furthermore, the points for manual pose estimation used with the NeuroPostureApp are illustrated in yellow. (**C**,**D**) The extracted mesh in the “Blender” software from a back and more frontal view for illustration. The algorithm used is not suitable to specifically estimate the hand position; therefore, these are not estimated correctly. LM, lateral malleolus.

We applied the Part Attention Regressor (PARE) method for 3D human body shape estimation on our photo archive of postural disorders that has already been used in the 2018 consensus on the measurement of the camptocormia angle.^2^ The PARE algorithm is a method that is robust to potential body occlusion in pictures. It is freely available for noncommercial scientific research purposes only. In short, the algorithm is based on convolutional neuronal networks that depict human shape and posture from common 2D pictures to fit a skinned multiperson linear model (SMPL) accordingly.^5^ The SMPL model is a 3D model of the human body that was constructed from 1786 high‐resolution 3D scans of humans in different postures.^6^ The network has been pretrained on large data sets of human posture. To measure the bending angle of the trunk, we followed the recommendations of the 2018 consensus, which assesses the angle between the connection of the spinous process of the vertebra C7 and L5 and, respectively, the lateral malleolus and the spinous process of L5 (please compare with Fig. 1). These anatomical landmarks were identified on the resulting 3D human model, which consists of 6890 points in total (see Supplementary Table S1). The estimated angles of the mesh‐based method were validated against the manual assessment of the flexion angle using the freely available NeuroPostureApp (http://www. neuroimaging.uni‐kiel.de/NeuroPostureApp), which was originally developed by our research group (see Fig. 1 and Video 1).

**Video 1:** The 3‐dimensional mesh recovery from common 2‐dimensional pictures for the automated assessment of body posture in camptocormia explained in a brief video

**FIG. 2:**
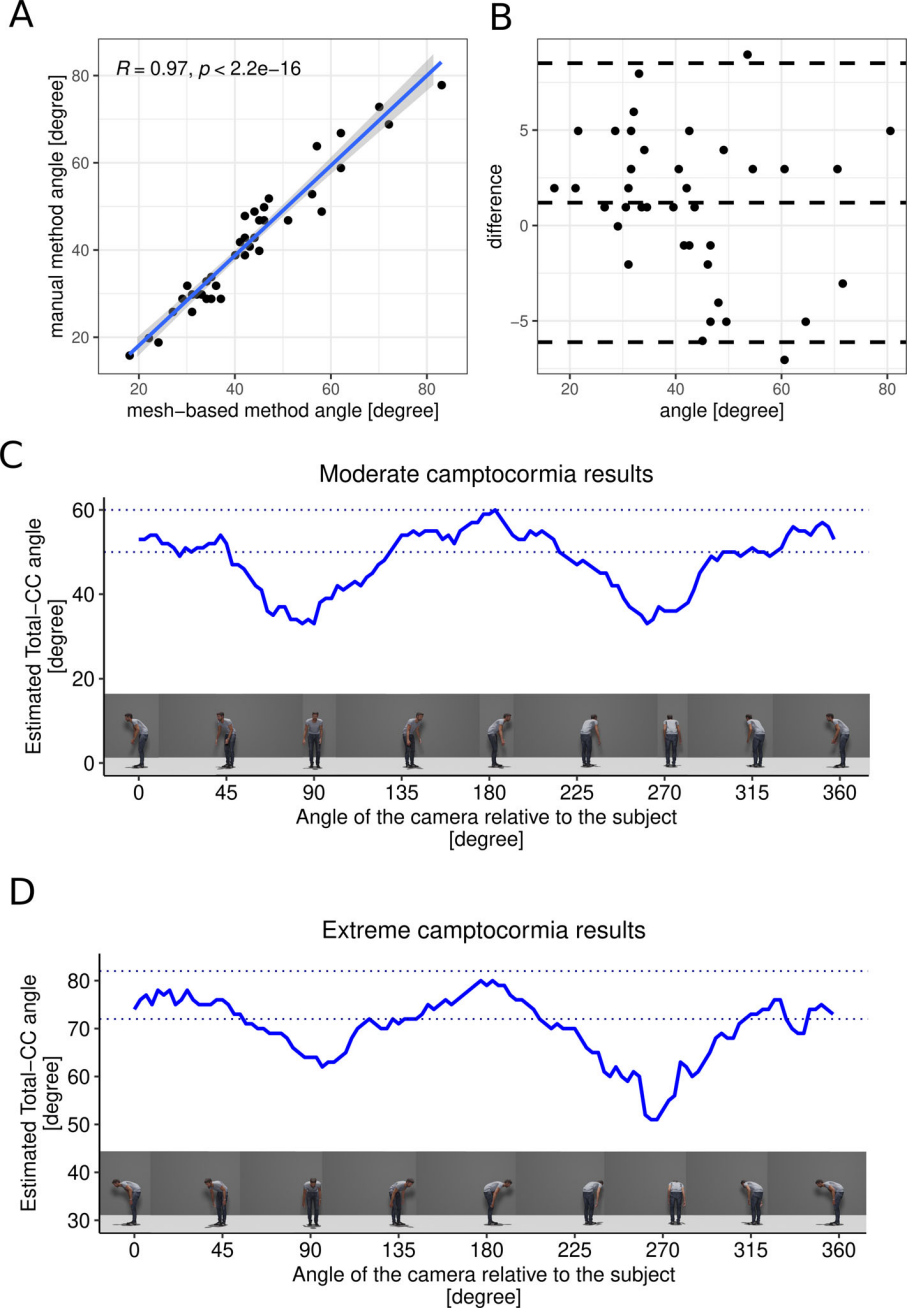
(**A**) Highly significant Pearson correlation between the angles of the trunk flexion measured in 40 pictures using the manual versus the automated mesh‐based method. (**B**) The associated Bland‐Altmann plot is shown. (**C**,**D**) Angle estimation on the virtual human posed in a moderate camptocormia posture (C) with a bending angle of 55 degrees measured by the manual method and an extreme camptocormia posture (D) with 77 degrees of forward flexion. The figure shows that the method does not provide reliable angle estimates if the image is created from an extreme front or rear perspective. The dark dotted lines indicate a range of 5 degrees above and below the manually measured value. CC, camptocormia.

As all photos of this data set were taken in strict lateral view, a second part of the study was designed to get an impression of the approach's measurement accuracy depending on the camera angle in relation to a subject. A simulated human body was positioned in 2 different postures: a moderate example of camptocormia and a very extreme case of camptocormia. For each of the 2 simulated bodies, pictures from 120 different camera positions were rendered using the open‐source software Blender.^7^ The camera rotated from 0 to 360 degrees in steps of 3 degrees starting and ending in a perfectly aligned lateral position (see Fig. 2).

A royalty‐free and rigged human 3D model was used. Correlation analysis and measures of variance were conducted using R.^8^

## Results

The clinical characteristics of the cohort are available in Supplementary Table S2. The comparison between the manual (angle, 43 ± 14 degrees; mean ± standard deviation [SD]) and the mesh‐based angle measurements (angle, 42 ± 15 degrees; mean ± SD) showed a significant Pearson correlation between both methods (*R* = 0.97, *P* < 0.05). The mean absolute error between methods with the manual as reference was 3.24 degrees (see Fig. 2B). The analysis of the rotated virtual body in 2 different camptocormia positions resulted in a stable performance of the angel assessment as the body is seen from a lateral perspective. As to be expected, as the view reaches a frontal or back position, the camptocormia angle estimation becomes unreliable.

## Discussion

This proof‐of‐principle study shows that the 3D model reconstruction from 2D‐RGB pictures offers promising applications for the estimation of postural disorders. The assessment of the camptocormia angle using the manual method and the mesh‐based method on our archive photo material resulted in a convincing correlation. Opposing the methods by Shin et al^3^ and Zhang et al^4^ that have been published on automated measurement of the camptocormia angle, the current recommendation of clinical experts to assess the camptocormia angle can be implemented with 3D mesh recovery methods.^2^ A major advantage over the classic method is that reliable results are achieved by the new method even with photos that are not taken from a perfect lateral perspective. Moreover, the recovered 3D model adds a further perspective for the clinician to retrospectively access a patient's posture more in detail. This allows a comprehensive analysis of other angles in the body shape (eg, knee and hip flexion), which have received little attention so far. Furthermore, the assessment of other postural disorders such as Pisa syndrome and antecollis can be possibly implemented and should be evaluated. However, for the possible application on videos, a more accurate validation against the gold standard, such as using camera‐based motion capture or internal measurement unit motion capture systems, is necessary, as the manual assessment of the flexion angle has not been validated under dynamic circumstances. This method is not unlimited, though. If the camera angle relative to a perfect lateral view exceeds more than approximately 40 degrees, the deduction of the bending angle becomes unreliable in the mesh‐based method as shown in Figure 2. Algorithms specifically trained on parkinsonian posture might enhance this approach's performance. Currently, 3D mesh recovery algorithms still require the above‐average computing power provided by graphic processing units. It is to be expected that further technical progress will lower the computing power threshold and certainly increase the estimation accuracy. In conclusion, this new approach is recommended for further research as a reliable and more robust method of angle detection in postural disorders because it is easier to use due to the possibility of automation and for the first time offers the possibility of a comprehensive analysis of body posture beyond the forward‐bending angle.

## Author Roles

(1) Research Project: A. Conception, B. Organization, C. Execution; (2) Statistical Analysis: A. Design, B. Execution, C. Review and Critique; (3) Manuscript Preparation: A. Writing of the First Draft, B. Review and Critique.

R.W.: 1A, 1B, 1C, 2A, 2B, 3A

N.G.M.: 2C, 3B

O. Gavriliuc: 2C, 3B

O. Granert: 2C, 3B

G.D.: 2C, 3B

## Disclosures

**Ethical Compliance Statement:** The study was approved by the ethics committee of the Medical Faculty of Kiel University. Informed written consent was obtained from all participants. We confirm that we have read the Journal's position on issues involved in ethical publication and affirm that this work is consistent with those guidelines.

**Funding Sources and Conflicts of Interest:** No specific funding was received for this work. The authors declare that there are no conflicts of interest relevant to this work.

**Financial Disclosures for the Previous 12 Months:** R.W. and O. Gavriliuc have nothing to declare. G.D. has served as a consultant for Cavion and Functional Neuromodulation. He has received royalties from Thieme publishers and funding by the German Research Council (Sonderforschungsbereich [SFB] 1261, T1). N.G.M. reports travel grants, lecture fees, and project support from Angelini Pharma, Jazz Pharma, Eisai Pharma, UCB Pharma, LivaNova, and Desitin Pharma. O. Granert worked as a part‐time software engineer for AIRAmed GmbH.

## Supporting information


**Table S1.** Clinical characteristics of the evaluated subjects.

## Data Availability

Due to privacy regulations, the data cannot be published or shared with third parties. The code is available at https://github.com/Wolkero/3dCCangle.
