# Supplementary material for: Three‐Dimensional Mesh Recovery from Common 2‐Dimensional Pictures for Automated Assessment of Body Posture in Camptocormia
Source: Mov Disord Clin Pract. 2023 Jan 24;10(3):472–6. doi: 10.1002/mdc3.13647 (PMC10026267; doi:10.1002/mdc3.13647)
Supplement: Supplementary file 1 — Table S1. Clinical characteristics of the evaluated subjects. [file MDC3-10-472-s001.docx]

**Supplementary material**

**Supplementary table 1.**

| Anatomical landmarks | SMPL vertex index* |
| --- | --- |
| spinous process C7 | 829 |
| spinous process L5 | 3021 |
| right lateral malleolus | 6728 |
| left lateral malleolus | 3327 |

*Vertex point on the SMPL mesh surface corresponding to the anatomical landmark

| *Age (years)* | *72.5 (7.13)** |
| --- | --- |
| *Sex (male/female)* | *28/13* |
| *Disease duration (years)* | *14.75 (8.0)** |
| *Hoehn & Yahr stage* | \| stage \| n \| \| --- \| --- \| \| 0 \| 0 \| \| 1 \| 0 \| \| 2 \| 2 \| \| 2,5 \| 1 \| \| 3 \| 18 \| \| 4 \| 15 \| \| 5 \| 3 \| \| NA** \| 2 \| |
| *UPDRS III* | *32.15 (15.12)** |
| *Daily Levodopa equivalent dose (mg)* | *886 (336)** |
| **mean (sd), **two patients suffered of MSA, the H&Y is not mentioned in this case.* | |

Supplementary table 1: Clinical characteristics of the evaluated subjects.
